# Poly(Glycerol Succinate) as an Eco-Friendly Component of PLLA and PLCL Fibres towards Medical Applications

**DOI:** 10.3390/polym12081731

**Published:** 2020-08-03

**Authors:** Dorota Kolbuk, Oliwia Jeznach, Michał Wrzecionek, Agnieszka Gadomska-Gajadhur

**Affiliations:** 1 Laboratory of Polymers and Biomaterials, Institute of Fundamental Technological Research, Polish Academy of Sciences, Pawińskiego 5B Street, 02-106 Warsaw, Poland; oliwia.jeznach@gmail.com; 2 Faculty of Chemistry of Warsaw University of Technology, Noakowskiego 3 Street, 00-664 Warsaw, Poland; michal.wrzecionek.dokt@pw.edu.pl

**Keywords:** poly(glycerol succinate), plasticiser, eco-friendly polymer, electrospinning, hyperbranched polyester

## Abstract

This study was conducted as a first step in obtaining eco-friendly fibres for medical applications using a synthesised oligomer poly(glycerol succinate) (PGSu) as an additive for synthetic poly(L-lactic acid) (PLLA) and poly (L-lactide-co-caprolactone) (PLCL). The effects of the oligomer on the structure formation, morphology, crystallisation behaviour, and mechanical properties of electrospun bicomponent fibres were investigated. Nonwovens were investigated by means of scanning electron microscopy (SEM), wide angle X-ray scattering (WAXS), differential scanning calorimetry (DSC), and mechanical testing. The molecular structure of PLLA fibres is influenced by the presence of PGSu mainly acting as an enhancer of molecular orientation. In the case of semicrystalline PLCL, chain mobility was enhanced by the presence of PGSu molecules, and the crystallinity of bicomponent fibres increased in relation to that of pure PLCL. The mechanical properties of bicomponent fibres were influenced by the level of PGSu present and the extent of crystal formation of the main component. An in vitro study conducted using L929 cells confirmed the biocompatible character of all bicomponent fibres.

## 1. Introduction

In the last few years, interest in new green hyperbranched polyesters (HBPEs) has grown rapidly. HBPE-derived renewable biomonomers are particularly suitable as plasticisers due to their simultaneous effectiveness and low migration from a polymer matrix [1]. HBPEs have attracted considerable attention due to their unique structures, possessing a large number of end groups, low viscosity, good solubility, and facile synthesis procedures [2]. The unusual plasticising properties of these materials largely arise from the presence of atomic-scale free-volume cavities [1]. Poly(glycerol succinate), poly(glycerol succinate-co-maleate), poly(glycerol adipate), poly(glycerol-co-diacid), and poly(glycerol sebacate) have been synthesised to support the development of new bio-based HBPEs for various applications [3,4,5,6].

**Figure 1:**
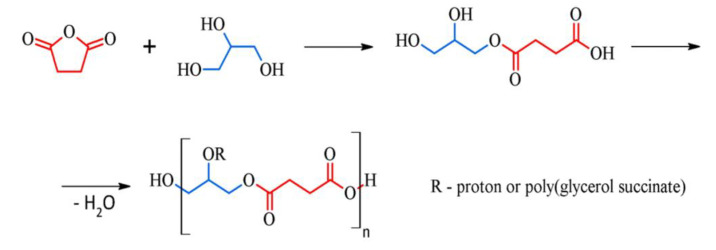
Reaction schematics of poly(glycerol) succinate (PGSu).

Poly(glycerol succinate) (PGSu) is one of the examples of polyesters obtained from glycerol and dicarboxylic acids such as adipic, sebacic, or succinic acid [7]. PGSu is synthesised from two bio-based monomers—glycerol and succinic acid (Figure 1). Glycerol is a major by-product of the biodiesel industry. In 2012, the production of bioglycerol reached over 2 million tonnes [8]. Succinic acid is a product derived from the fermentation process of biomass resources, and its production has also been developed on an industrial scale [9]. To synthesise biodegradable polyesters from glycerol and succinic acid, bulk polycondensation in the absence of a solvent and a catalyst is used. Moreover, no toxic waste materials are produced during the reaction. PGSu macromolecules can form dendrimers, branched, highly branched, and hyperbranched polymers [10]. Synthesis conditions have a significant impact on the physical and mechanical properties of HBPEs due to differences in molecular weight, cross-linking density, and degree of branching. The obtaining of liquid or gelated form depends on the molar ratio of reactants determining the ratio of hydroxyl to carboxylic groups [11].

Poly(glycerol succinate) has been used as an accelerant for poly(caprolactone) degradation enabling moisture penetration to the hydrophobic matrix [6]. Hydrophilic PGSu oligomers can be introduced as polar head groups into surfactants after grafting fatty alkyl chains, leading to amphiphilic structures. PGSu might be suitable as a bio-based surfactant for various products (e.g., shampoos, body washes, kitchen cleaners) to minimise the damage that synthetic detergents can cause to skin and hair [1]. PGSu has the same similarities with another glycerol-based polyester, poly(glycerol sebacate), which is widely known in the field of tissue engineering and was proposed as a scaffold material or scaffold additive for cardiac, bone, cartilage, nerve, and corneal tissues [12,13].

Poly(L-lactic acid) is an amorphous or semicrystalline polyester produced by the condensation of lactic acid obtained from renewable resources. It is commonly used in biomedical applications and displays functional properties comparable to those of petroleum-based polymers [14]. High tensile strength and elastic modulus are characteristic for this polymer. On the other hand, this polymer is relatively brittle and has low values of elongation at break and low impact resistance [5,15]. Hence, there is a need to modify the properties of poly(L-lactic acid) (PLLA). One approach is copolymerisation with caprolactone. Poly (L-lactide-co-caprolactone) (PLCL) displays much higher elasticity in comparison to that of pure PLLA and is widely used in biomedical applications.

There are a few examples of the utilisation of glycerol-based HBPEs as modifiers for PLLA. These materials behave as plasticisers and increase chain mobility. Blending PLLA with plasticisers is one of the most effective approaches for toughening PLLA [16]. The addition of 15% poly(glycerol) sebacate to PLLA has been seen to increase the elongation at break from 7% to 155% [17]. Polyesters from glycerol, sebacic acid, and stearic acid have been synthesised and used to modify PLA [18]. Elongation at break has been increased from 2.3% for native PLLA to 93% for PLLA with 10% addition of modifier. Poly(glycerol) succinate and its copolymer with maleic anhydride have been used as toughening agents [3,5]. For example, PLLA was blended with PGSu at a weight ratio of 80/20 during an extrusion process. Particles of PGSu (6−15 µm in diameter) were dispersed within the PLLA matrix. As was expected, modification caused an increase of elongation at break (from 4% to 11%). Simultaneously, tensile strength and modulus decreased by 40% and 26%, respectively. The improvement of elongation at break is associated with molecular bonding interactions and entanglements between PGSu and PLLA, which are influenced by their molecular weights and conformations. Moreover, the behaviour of plasticisers as additives in amorphous polymers may be different from that for semicrystalline polymers [15,19]. In amorphous polymers, oligomer additives play the role of plasticisers and increase elongation and tensile strength, while in semicrystalline polymers, phase separation contributes to the inefficient impact on mechanical properties [20].

Previous reports on electrospun PGSu or blends of PGSu with polyesters are not available. In this work, the structure, crystallisation, and mechanical properties of amorphous PLLA and semicrystalline PLCL blended with PGSu were investigated. An electrospinning process was used to fabricate bicomponent fibres. Electrospun nanofibres were characterised using SEM, wide angle X-ray scattering (WAXS), differential scanning calorimetry (DSC), and tensile testing in order to explain the effects of PGSu on fibre properties. The effect of the presence of a PGSu additive on molecular mobility for the amorphous PLLA and semicrystalline PLCL was analysed. Finally, the biocompatibility of all bicomponent fibres was confirmed using in vitro testing.

## 2. Experimental

### 2.1. Oligomer Poly(Glycerol Succinate)

#### 2.1.1. Synthesis

Poly(glycerol succinate) (PGSu) synthesis was carried out in the Warsaw University of Technology (Warsaw, Poland). Briefly, succinic anhydride (0.3 mol; 30.00 g; ≥97% Fluka, Munich, Germany) and glycerol (0.3 mol; 27.6 g; ≥99% Sigma Aldrich, Saint Louis, MO, USA) were used as monomers in nonsolvent polycondensation. The synthesis was carried out in the MultiMax reactor system produced by Mettler Toledo, Columbus, OH, USA. The reaction system comprised a glass reactor equipped with a mechanical stirrer, a temperature sensor, and a reflux condenser. The reaction mixture was heated up to 150 °C; then, the process was continued for 4 h. After completion of the synthesis, the product was purified by rotary distillation under a pressure of 10 mbar and at a temperature of 40 °C.

#### 2.1.2. Chemical Structure

A Fourier transform infrared spectroscopy (FTIR) (Alpha II BRUKER, Karlsruhe, Germany) analysis was performed to study the molecular structure of PGSu. Measurements were carried out using the attenuated total reflectance (ATR) technique. For each sample, 32 scans in the range 400–4000 cm^−1^ were performed and averaged.

Nuclear magnetic resonance (NMR) spectroscopy was used to confirm the ester structure and to designate poly(glycerol succinate) molecular weight according to the methodology used previously [6]. Briefly, 200 mg of poly(glycerol succinate) was dissolved in deuterated methanol (1 mL) for 24 h, and then, the solution was transferred to an NMR tube. ^1^H NMR spectres were obtained using a Merkury-400BB spectrometer (400 MHz) Varian, Inc., Palo Alto, CA, USA). As a result of the well-resolved signals from the succinic part, they are used to determine the molar mass of the obtained polyester. For this reason, 2.61 ppm (2H, acid end)—A and 2.67 ppm (4H, ester)—B signals were integrated and used. Molar weight was calculated according to the following formula:

(1) $$M_{NMR}=M_{0}\times \frac{\int B}{2\int A}+18$$

where *M_NMR_*—molecular weight was calculated based on NMR, *M*_0_—repetitive unit molecular weight (192 Da), ∫A—integral of *A* signal, ∫B—integral of *B* signal.

In addition, the degree of branching (*DB*) was determined based on the ^1^H NMR spectrum. The glycerol metin proton in triester (dendrimer) has a characteristic chemical shift of 5.08 ppm—D. Again, we decided to compare it to the well-resolved signal from the succinic diester part, which represents each repetitive unit. *DB* was calculated via the following formula:

(2) $$DB=\frac{4\int D}{2\int A}$$

where ∫A—integral of *A* signal, and ∫D—integral of *D* signal.

The acid number (AN): First, 0.1 g of the sample was weighed out on an analytical balance (RADWAG XA 160/2X, Radom, Poland) and then dissolved in a methanol/water mixture (1:9). The samples were titrated (in 0.1 M aqueous solutions of sodium hydroxide) potentiometrically (716 DMS TitrinoMetrohm automatic burette and Hydromet potentiometric electrode), which allowed for the exact determination of endpoints for the individual components of the post-reaction mixture. The acid number was calculated according to the following formula:

(3) $$\mathrm{AN}=\frac{56.1\times V\times M_{\mathrm{NaOH}}}{m}$$

where AN—acid number, 56.1—the molar mass of KOH (mg/mmol), V—titrant volume for titration of the polymer only, n—KOH solution titer, and m—the mass of the analysed sample.

The ester number (*EN*): First, 0.2 g of the sample was weighed out on an analytical balance; then, 15 mL of methanol and 20 mL of 0.1 M aqueous KOH solutions were added. This mixture was refluxed in a water bath for 1 h. After cooling, the solutions were titrated in the presence of phenolphthalein with 0.1 M hydrochloric acid. A blank test was carried out simultaneously. EN was determined based on the following formula:

(4) $$\mathrm{EN}=\frac{\left(V_{0}-V\right)\times M_{\mathrm{HCl}}\times 56.1}{m}-\mathrm{LK}$$

where V—the volume of titrant used for titration of the sample, V_0_—the volume of titrant used for titration of the blank sample, M_HCl_—titer of HCl (titrant) used for titration, 56.1—the molar mass of KOH, m—the sample mass, and AN—acid number.

For both AN and EN determinations, the tests were done in triplicate. For the ED calculation, an averaged result of AN and EN was used.

The esterification degree (ED): based on the acid number and ester number, ED was determined, according to the following formula:

(5) $$\mathrm{ED}=\frac{\mathrm{EN}}{\mathrm{AN}+\mathrm{EN}}$$

Based on the esterification degree, the number average molecular weight M_n_ and weight average molecular weight M_w_ were calculated according to previously reported formulas [6].

### 2.2. Bicomponent Fibres Fabrication

#### 2.2.1. Materials

Commercial polyesters: poly(lactide) PL49 (Corbion, The Netherlands) and poly(lactide-co-caprolattone) LC703S (Evonic, Niderlands) were used as the main components of electrospun bicomponent fibres. PGSu was synthesised according to the methodology described above. As a solvent for mono- and bicomponent fibres 1,1,1,3,3,3-hexafluoro-2-propanol (HFIP) (Iris Biotech GmBH, Marktredwitz, Germany) was used. Polymers in a specific ratio of PGSu (2–40%) to PLLA or PLCL (*w/w*) were dissolved in HFIP at concentrations of 3.5% and 8% (*w/w*), respectively (Table 1). The distances between both needles and the collector were 15 cm, the flow rate of the solution on both sides was 1.5 m/h, and the inner diameter of the needles was 0.34 mm. For most types of electrospun solutions, a voltage in a range of 13–13.5 kV was used to obtain uniform fibres and to maintain a stable electrospinning process. All nonwovens were electrospun at a similar temperature (22–24 °C) and humidity (45–55%). After electrospinning, all nonwovens were placed under a fume hood to ensure no residual solvent remained in the fibres before the next experimental steps. Sample acronyms are shown in Table 1.

#### 2.2.2. Structure and Morphology

Fibre morphologies were characterised using a scanning electron microscope (SEM) (JSM-6010PLUS/LV InTouchScope™Jeol, Tokyo, Japan) at an accelerating voltage of 5 kV. All samples were coated with gold before taking SEM observations. The fibre diameter was determined as the average value from 100 measurements for each nonwoven using ImageJ software and SEM images.

The crystal structure and crystallinity of PLLA in electrospun PLLA, PLCL, and bicomponent fibres with PGSu were characterised by wide-angle X-ray scattering (WAXS) (D8 Discover diffractometer, Bruker, Germany). Radial profiles were recorded using CuKα radiation, λ = 1.5418 Å in coupled theta—2theta mode. In this type of scan, divergent beam optics were used, with 1 mm primary linear slit and axial divergence Soller 2.5° on the primary beam side. On the secondary beam side, axial divergence Soller 2.5° was used as well as an Ni filter to exclude a Cu_Kβ_ component. The results were initially analysed using Bruker evaluation software. The “empty” scan (a scan of the measuring table without sample) was subtracted, and the default function of subtracting the background was applied.

The amorphous halo peaks and the mesophase peak in WAXS profiles were identified based on the fitting method outlined by Monnier et al. [19]. The weight fraction of the various phases was computed from the ratio of the specific scattering contribution of each one to the total scattering area. The spectrum of pure PGSu was determined as a reference.

Characteristic temperatures, phase transition enthalpies of pure PLLA, PLCL, and bicomponent fibres with PGSu were investigated using differential scanning calorimetry (DSC, Perkin-Elmer Pyris-1 apparatus, Waltham, MA, USA). Samples weighing ca. 2 mg were heated at a rate of 10 °C/min in a range from −50 to 200 °C. Gas nitrogen was used as a protective medium during measurements.

The mechanical properties of the materials were measured by a uniaxial testing machine (Lloyd EZ-50 equipped with handles for thin and delicate samples) with a 50N load cell under a cross-head speed of 10 mm/min (Lloyd Instruments Ltd, Bognor Regis, United Kingdom). Three 10 mm × 50 mm samples (10 mm × 25 mm outspread between handles being subjected for testing) were prepared for each material type. Their thickness ranged from 50 to 80 µm, which was measured for each sample and included in further analysis. From the stress–strain curves, Young’s modulus, tensile strength, and elongation at break were determined. All measurements were performed on dry samples.

#### 2.2.3. Cellular Viability

Biological studies were performed using mouse fibroblasts. Tests were done using a previously developed procedure [21,22], which is briefly described below.

*Cell cultures:* L929 cells were cultured on 25 cm^2^ flasks in a culture medium and held in an incubator. Cells were removed from the flasks using 0.05% trypsin. Then, the harvested cells with an addition of 7 mL of culture medium were centrifuged for 5 min at 100× *g*. The collected pellet was dispersed in the culture medium, and the cell amount was measured using a TC2 Automated Cell Counter Bio-Rad.

*Sample preparation:* All materials were cut into disks of nonwovens with a 10 mm diameter, which is suitable for 48 well plates. Samples were sterilised in UV light for 30 min on each side.

*Cellular viability in contact mode determined in Presto Blue test:* The cellular viability of cells seeded on samples was evaluated. L929 cells in a density of 3 × 10^3^ cells/well were seeded into a 48-well plate with bottoms lined with nonwovens and put in an incubator for few days. After 3 and 7 days of in vitro study, each well was filled with 180 µL of media and 20 µL of Presto Blue reagent and kept in the incubator for 60 min. After this step, 100 µL from each well was transferred to a 96-well plate. Fluorescence was measured using a FLUOstar Omega apparatus (Ortenberg, Germany).

#### 2.2.4. Statistical Analysis

In most experiments, at least three samples for each kind of material were tested (excluding WAXS, DSC). The presented data were calculated as average values. Student’s *t*-test was used to determine the statistical significance between the data.

## 3. Results

### 3.1. Oligomer PGSu

**Figure 2:**
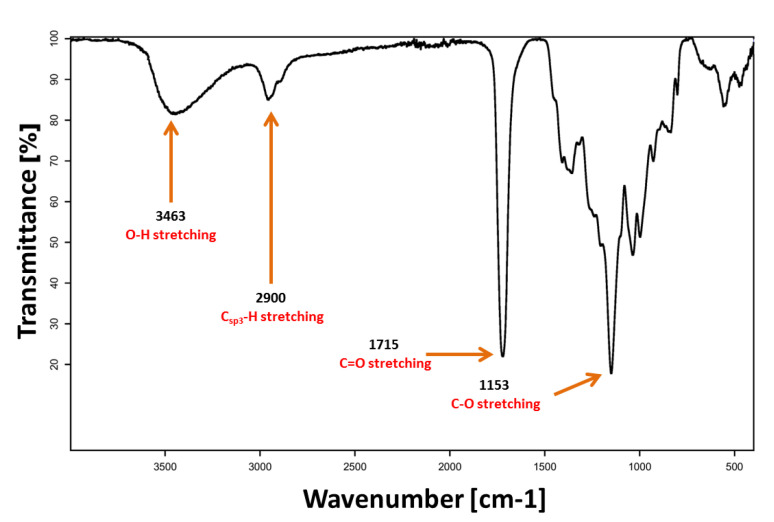
Fourier transform infrared spectroscopy (FTIR) spectra of PGSu.

The oligomer of PGSu was synthesised in a nonsolvent polycondensation process according to the specific methodology described above. According to Figure 2, the wide band at 3463 cm^−1^ was attributed to the O–H stretching, the wide band with two vertices with the centre at 2900 cm^−1^ was assigned for the C_sp3_–H stretching, the sharp band at 1715 cm^−1^ was attributed to C=O stretching, and the band at 1153 cm^−1^ was assigned for the C–O stretching. FTIR analysis confirms evidence of the chemical groups’ characteristics in the PGSu structure. Bands at 1715 cm^−1^ and 1153 cm^−1^ show the existence of ester bonds in the oligomer structure. The band at 3463 cm^−1^ confirms the existence of H-bond associates due to termination of the polymer by hydroxyl and carboxyl groups. There are not any characteristic bands of the succinic anhydride monomer in the spectra.

**Figure 3:**
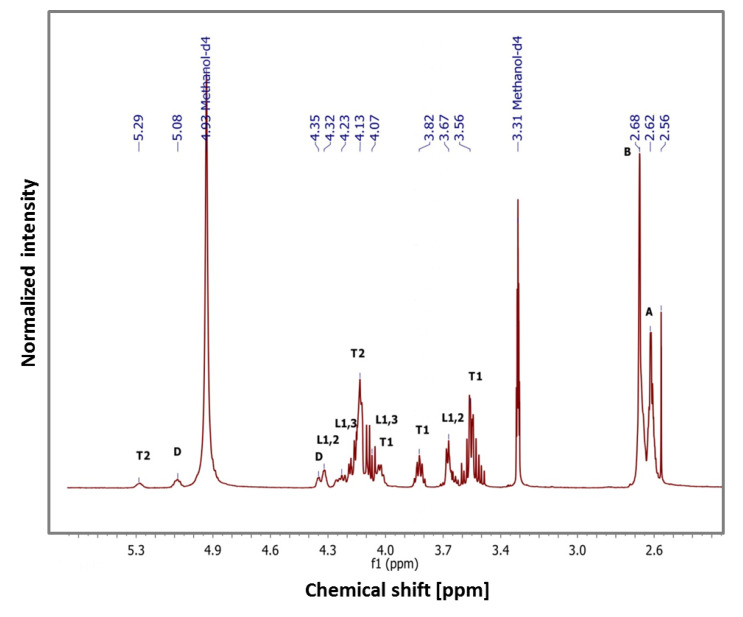
^1^H NMR spectra of PGSu (400 MHz, methanol-d_4_) and signals characteristic for the PGSu structure: succinic end group (A), succinic ester (B), succinic acid (C), glycerol spin systems (D, T1, T2, L1.2, L1.3).

Figure 3 illustrates the ^1^H NMR spectra of PGSu with an interpretation of proton signals. The signal at 2.62 (A) is related to the succinic end group (–C**H_2_**–COOH), the signal at 2.68 ppm (B) is related to the succinic ester (–OOC–(C**H_2_**)_2_–COO–), the signal at 2.56 ppm is related to the amount of succinic acid (HOOC–(C**H_2_**)_2_–COOH), while the signals above 3.5 ppm marked as D, T1, T2, L1.2, L1.3 were related to glycerol spin systems, which were previously described for poly (glycerol sebacate) [6].

Based on ^1^H NMR, the molecular mass M_NMR_ of PGSu oligomers was determined as c.a. 786 Da., the PGSu acid number was determined as 151.1 mg KOH/g, and the ester number was 429.6 mgKOH/g, which results in *ED* = 0.74. Due to this number, the average molecular weight *M*_n_ equals 739 Da and the weight average molecular weight *M*_w_ 1285 Da. The PGSu DB calculated based on this spectra is c.a. 0.25.

### 3.2. Bicomponent Fibres

#### 3.2.1. Morphology and Structure

**Figure 4:**
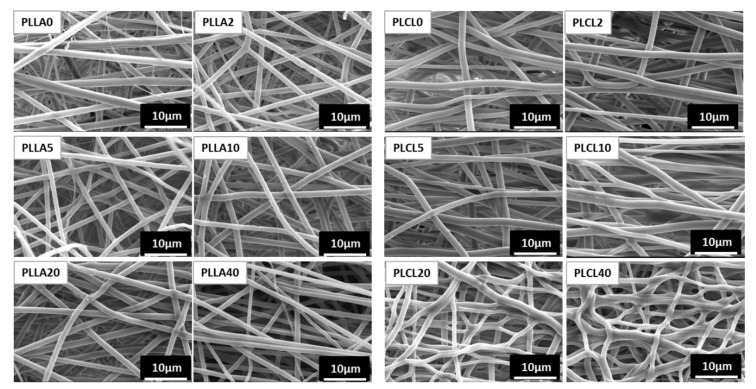
Morphology of electrospun bicomponent fibres with PGSu additive: PLLA2, PLLA5, PLLA10, PLLA20, and PLLA40 in relation to PLLA0 (pure PLLA), and PLCL2, PLCL5, PLCL10, PLCL20, and PLCL40 in relation to PLCL0 (pure PLCL).

PGSu was mixed with different amounts of PLLA or PLCL, and bicomponent fibres were formed in an electrospinning process (Table 1). Fibre morphology was illustrated by SEM and presented in Figure 4. All fibres are free of beads; only the PLCL20 and PLCL40 fibres indicate a slightly nonuniform morphology (Figure 4).

**Figure 5:**
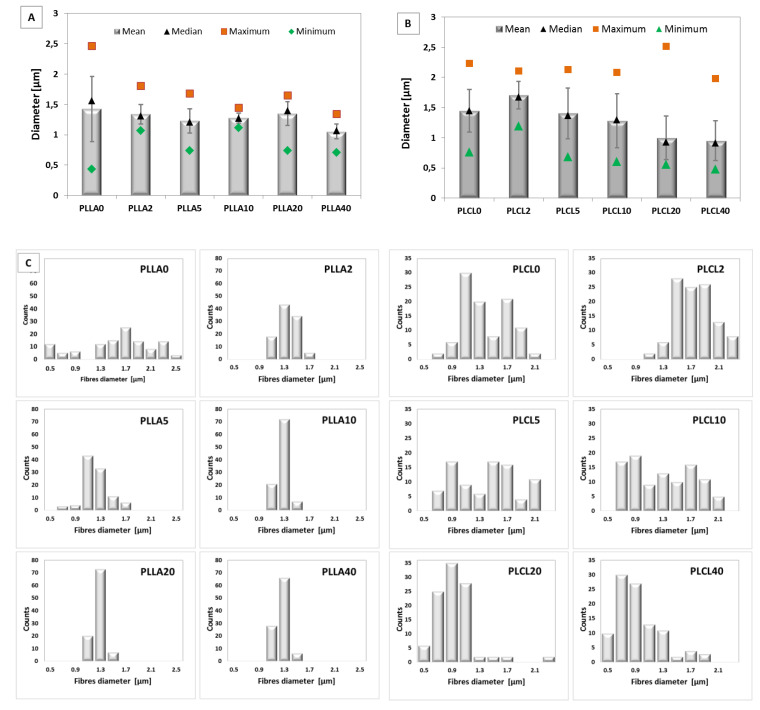
Diameter of electrospun bicomponent fibres with PGSu additive: (**A**) PLLA2, PLLA5, PLLA10, PLLA20, and PLLA40 in relation to PLLA0 (pure PLLA); (**B**) PLCL2, PLCL5, PLCL10, PLCL20, and PLCL40 in relation to PLCL0 (pure PLCL) determined with SEM images; (**C**) histograms of all fibre diameters.

The fibre diameter was determined from SEM images and is illustrated in Figure 5A,B. Mean diameter, and median, maximum, and minimum diameters were calculated. Both types of bicomponent fibres indicate a mean fibre diameter below 1.5 µm. What is more, the fibre diameter slightly decreases with the PGSu additive in relation to the pure polyesters PLLA and PLCL. Histograms of dimeter distribution indicated the unimodal character of the bicomponent fibres of PLLA with PGSu (Figure 5C). While PLL0, PLCL0, and bicomponent fibres of PLCL with PGSu indicated a bimodal distribution of the diameter of the fibres.

**Figure 6:**
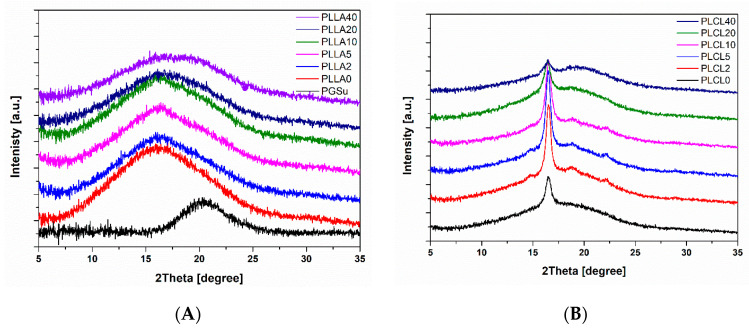
Wide angle X-ray scattering (WAXS) spectra of electrospun bicomponent fibres with PGSu additive: (**A**) PLLA2, PLLA5, PLLA10, PLLA20, and PLLA40 in relation to PLLA0 (pure PLLA); (**B**) PLCL2, PLCL5, PLCL10, PLCL20, and PLCL40 in relation to PLCL0 (pure PLCL).

The WAXS spectra of all electrospun bicomponent fibres in relation to pure PGSu, PLLA, and PLCL are illustrated in Figure 6A,B. PGSu was fully amorphous (Figure 6A). PLLA fibres indicate three peaks from amorphous phases located at 2θ = 15.1°, 2θ = 21.5°, and 2θ = 30° and the amorphous peak from PGSu located at 2θ = 20.4 [20,23,24]. An increase of PGSu additive to PLLA up to 5% and 10% (PLLA5, PLLA10) contributes to a slight increase of molecular order, which might be noticed as a peak at 2θ = 16.1° related to the mesosphere [20].

The WAXS profile of PLCL indicates single peaks at 2θ = 16.6° (Figure 6B) corresponding to the (110)/(200) lattice planes and broad amorphous halo with a maximum at 17.1° [25]. Selected bicomponent fibres, PLCL2, PLCL5, and PLCL10, indicate additional peaks at 2θ = 14.7°, 19°, and 22.4° (Figure 6B) corresponding to the (010), (203), and (015) lattice planes, respectively [24].

**Figure 7:**
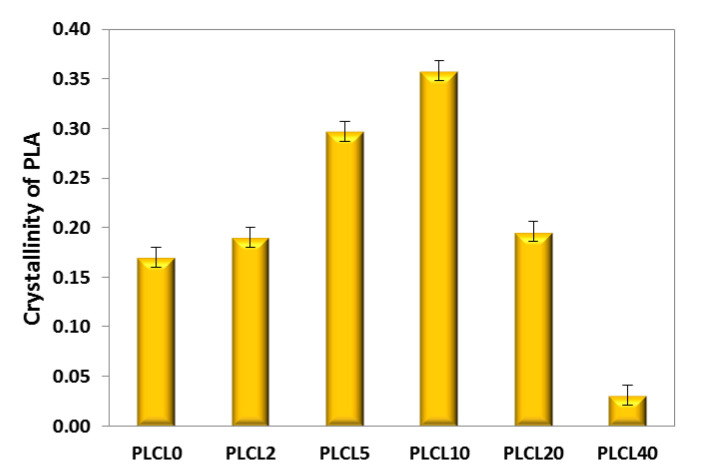
Crystallinity of electrospun bicomponent fibres with PGSu additive: PLCL2, PLCL5, PLCL10, PLCL20, and PLCL40 in relation to PLCL0 (pure PLCL) determined with WAXS spectra.

Fibres formed from PLLA with PGSu indicate a zero amount of crystal phase, and the mesophase amount did not exceed 5%. Bicomponent fibres formed from PLCL indicate a local increase of crystallinity with the PGSu additive (Figure 7). The degree of crystallinity changed from 17% (pure PLCL) to 35% for PLCL10; then, it decreased to 19% and 3% for PLCL20 and PLCL40, respectively. A DSC analysis allows the determination of enthalpies of relaxation, cold crystallisation, and crystal phase melting, as well as characteristic temperatures of relaxation, cold crystallisation, and melting. Changes in the characteristic temperatures of bicomponent fibres in relation to PLLA and PLCL were observed. PLLA indicates an endothermic peak related to structural relaxation with a maximum level of c.a 55 °C, an exothermic peak as a result of cold crystallisation with a maximum of 67 °C, and a final crystal phase melting peak with a maximum of c.a. 181 °C. Between cold crystallisation and melting, the peak extends to a wide halo related to crystallisation during heating. The oligomer of PGSu indicates *T*_g_ at −38.13 °C, while it indicates PLLA at 50.87 °C. Bicomponent PLLA fibres indicate a double T_g_ characteristic for PGSu and PLLA. However, those temperatures are shifted towards each other in samples with a PGSu additive in relation to pure PLLA, as can be seen in Table 2. Additionally, as is indicated in Table 2, a PGSu additive in bicomponent PLLA fibres blocks relaxation. Thus, the relaxation temperature of PLLA increases, while the enthalpy of relaxation of PLLA decreases with the PGSu amount. In addition, there is a higher cold crystallisation capacity of PLLA in the presence of PGSu up to 10% (w/w) and slightly higher temperature of cold crystallisation. This corresponds to an increase of enthalpy of crystal phase melting with a small amount of PGSu additive to PLLA (PLLA2) and a slightly changed melting temperature Tm.

Tg of PGSu in bicomponent fibres with PLCL increased significantly (Table 2). The DSC spectra of PLCL indicate a single peak correlated with crystal phase melting, with the maximum at 157.24 °C (Table 2). The enthalpy of crystal phase melting of PLCL was 24.89/g, and it increased with the PGSu additive while maintaining the maximum peak position (Table 2).

#### 3.2.2. Mechanical Properties

**Figure 8:**
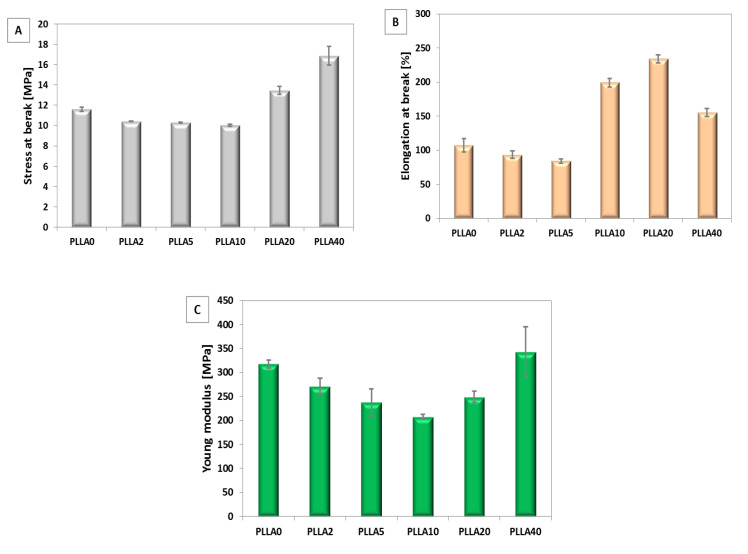
Mechanical properties of bicomponent fibres with additive PGSu to PLLA: (**A**) stress at break, (**B**) elongation at break, and (**C**) Young’s modulus.

**Figure 9:**
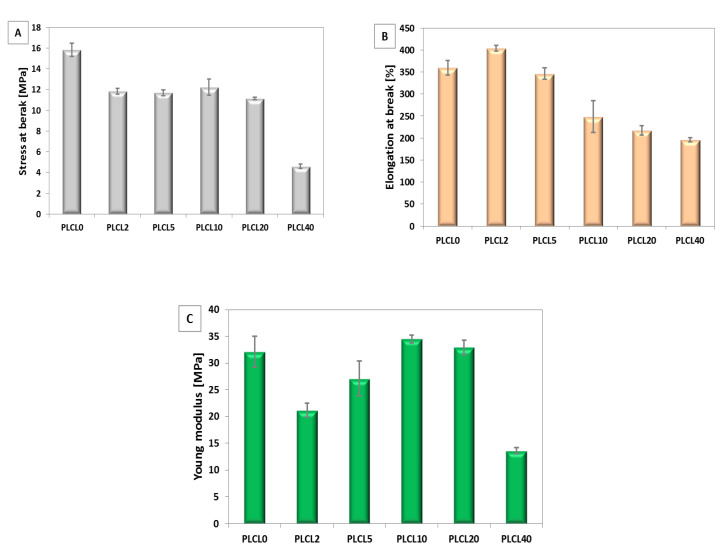
Mechanical properties of bicomponent fibres with additive PGSu to PLCL: (**A**) stress at break, (**B**) elongation at break, and (**C**) Young’s modulus.

The mechanical properties stress at break, elongation, and Young’s modulus of electrospun fibres were determined and illustrated in Figure 8A–C and Figure 9A–C. In the case of bicomponent fibres with PLLA, stress at break increased significantly for samples with the highest amount of PGSu (PLLA20 and PLLA40). Elongation at break changes from 100% for pure PLLA0 to 240% for PLLA20. A decrease of Young’s modulus of PLLA was observed with PGSu additive. A minimum of Young’s modulus was observed for PLLA10.

Trends in changes of mechanical properties differ significantly between bicomponent fibres formed from PLLA in relation to PLCL. Elongation at break increases only for PLCL2 in relation to PLCL0. Other bicomponent PLCL fibres indicate a decrease of stress at break, elongation at break, and Young’s modulus in relation to PLCL0.

#### 3.2.3. Cellular Viability

**Figure 10:**
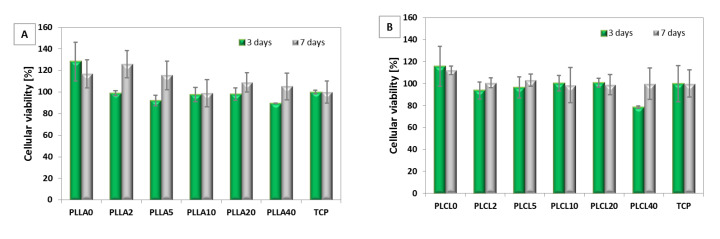
Cellular viability test on L929 cells after 3 and 7 days of cultivation on: (**A**) PLLA2, PLLA5, PLLA10, PLLA20, and PLLA40 in relation to PLLA0 (pure PLLA); (**B**) PLCL2, PLCL5, PLCL10, PLCL20, and PLCL40 in relation to PLCL0 (pure PLCL).

A viability test in contact mode indicated a slightly lowered cellular response after 3 and 7 days for bicomponent fibres in comparison to the PLLA, PLCL nonwovens, and tissue culture plastic (TCP). However, none of them proved to be cytotoxic (more than 70% of control) (Figure 10A,B). No effects of the molecular structure and mechanical properties of nonwovens were observed on cellular response.

## 4. Discussion

The oligomer of PGSu was successfully synthesised using a catalyst-free method combined with a polycondensation method. Characteristics of the PGSu bonds were confirmed by FTIR. Bands at 1715 cm^−1^ and 1153 cm^−1^ show the existence of ester bonds, and a band at 3462 cm^−1^ is evidence of H-bond associates, which is in line with the findings of other authors [6,7]. As the molar ratio of glycerol to succinic acid is increased in the PGSu products, the peak related to H-bonds becomes more visible, suggesting that the polyesters are hydroxyl-terminated [7]. Based on 1H NMR data, synthesised PGSu indicates characteristic groups and a hyperbranched structure with a 0.25 degree of branching (DB) with a molecular weight of M_NMR_ c.a 786 Da, which is typical for HBPEs [1,2,26]. Based on the esterification group, the number average molecular mass M_n_ of PGSu was equal to 739 Da, while M_w_ was equal to 1285 Da.

In this work, for the first time, we formed bicomponent fibres fabricated from PLLA and PLCL with different contents of PGSu using an electrospinning technique, and we investigated their morphology, structure, crystallisation behaviour, and mechanical properties. The diameter of all bicomponent fibres slightly decreased with the PGSu content due to the low molecular weight of PGSu and diminution of the final viscosity of the solution. The same effect of the decrease of diameter was observed for PLLA fibres with other oligomer additives [27]. The low molecular mass of plasticisers or other biological molecules contributed to a slight decrease of average molecular mass [28,29].

PGSu enhanced the segmental mobility of PLLA chains when its amount was above 20% (PLLA20, PLLA40), and it was the one of the reasons for the decreased T_g_ of PLLA that was observed previously [30]. Oligomer chains occupy intermolecular spaces between polymer chains, reducing the energy for molecular motion and the formation of hydrogen bonding between the polymer chains, which in turn increases the free volume and molecular mobility [31]. The initial rigid character of PLLA chains caused by high molecular weight contributes to the relatively high T_g_ of PLLA in bicomponent fibres. The higher T_g_ of PGSu in blends suggest changes in the mobility of oligomer chains, which might be related to good oligomer distribution [32] and/or the compatibility of both components. The T_g_ shift of the bicomponent fibre components towards each other observed in PLLA with PGSu (PLLA10, PLLA20) indicates polar interactions and compatibility between both polymers [4,33]. Mesophase formation in bicomponent fibres with PLLA was detected with WAXS, which proves its better chain mobility due to the PGSu additive.

PLLA is a fully amorphous, brittle polymer, so the amorphous phase arrangement plays a critical role during the PLLA tensile-strength test [17]. Young’s modulus defines elastic behaviour, and it is proportional to the ability of the amorphous chain to stretch in the direction of the applied force [34]. Mobility of the pure PLLA chains was low, so the Young’s modulus was relatively high with a value of 300 MPa. Under electrospinning conditions, the molecular orientation of all molecules increases due to applied voltage and shearing forces [18,35]. PGSu increased the chain mobility and cohesion of PLLA in bicomponent fibres, which has been observed previously using other plasticisers [36]. Those factors contribute to a reduction of the Young’s modulus and elongation at break, maintaining tensile strength of the bicomponent fibres with a PGSu component below 5% (w/w). Bicomponent nonwovens above 20% PGSu indicated tensile strength improvement due to the strength of bonds between mesophase chains and the appearance of hydrogen bonds in samples with a high PGSu amount, as was explained by Mangavel et al. [30], who analysed PLLA with various plasticisers. Hydrogen bonds could appear between the carbonyl oxygen and a proton from a free hydroxyl or carboxyl group. Regarding sustainability, the 10–20% (*w*/*w*) of the PGSu additive seems to be a perfect plasticiser of PLLA due to its biodegradable and eco-friendly character.

PLCL indicates semicrystalline character, so both phases—amorphous and crystalline—play a significant role in the molecular order, thermal properties, and mechanical properties. The T_g_’s of PGSu in blends with PLCL indicate the highest compatibility in PLCL5 where *T*_g_ = −15.98 °C. Other blends indicate T_g_ = −20.56 °C, −25.20 °C, and −30.96 °C for PLCL10, PLCl20, and PLCL40, respectively. We do not observe T_g_ from PLCL, which might be due to its full miscibility or signals overlapping. Pure PLCL indicate T_g_ at 38.12 °C, while in this region, there is a start of PCL melting and PLLA relaxation. The initial low molecular weight of PLCL0 in comparison to PLLA0 contributes to the relatively high mobility of PLCL chains. The T_g_ shift of the bicomponent fibre components towards each other observed in PLCL with PGSu indicates compatibility between both polymers [33,37,38,39,40]. The lower T_g_ of PGSu in blends might be related with good oligomer distribution also [32]. The crystallinity degree increased for PLCL/PGSu blends with a maximum for PLCL10 due to increased chain mobility. PGSu plays the role of nucleation agent, especially in blends with the lowest T_g_: PLCL5 and PLCL10, where chain mobility is high. In comparison to PLLA0, PLCL0 indicates higher chain mobility due to exposure to different chemical groups and a 2-fold lower *M*_w_.

Our pure PLCL indicates a 10-fold lower Young’s modulus than PLLA. Here, 2% of PGSu (PLCL2) was added without a nucleation effect. In this case, PGSu was located between amorphous phase molecules, so the Young’s modulus decreased with an increase in chain mobility. The Young’s modulus of bicomponent PLCL fibres correlated with crystallinity increased when the amount of PGSu was equal to or exceeded 5%. This phenomenon was related to the lower mobility of chains due to bonds occurring in the crystal phase and entanglement of chains of both components. Elongation at break dropped nearly two-fold with the PGSu additive due to the large variation in the phase composition (PLCL20, PLCL40). A low amount of PGSu (2–5%) contributed to the decrease of tensile strength because the plasticiser penetrated between the polymer chains and decreased the intermolecular forces, which caused lower polymer chain cohesion and easier elongation [13]. The degree of crystallinity can have a significant influence on the mechanical properties because it affects the extent of the intermolecular secondary bonding [34]. For crystalline regions in which molecular chains are closely packed in an ordered and parallel arrangement, extensive secondary bonding ordinarily exists between adjacent chain segments. This secondary bonding is much less prevalent in amorphous regions, by virtue of the chain misalignment. As a consequence, for semicrystalline polymers, tensile modulus increases significantly with the degree of crystallinity [34]. However, in the bicomponent fibres of PLCL and PGSu, this effect was not observed, and tensile at break decreased from c.a. 15 MPa to 11 MPa for PLCL2, PLCL5, PLCL10, and PLCL20. This might be explained as phase separation provoked by the crystallisation of the polymer and increased content of the plasticiser in the amorphous phase that leads to premature failure [41]. What’s more, Keith et al. reported that during crystallisation, impurities rejected by the growing crystallites tend to diffuse away radially from the spherulites rather than accumulate between the lamellae [42]. Spherulites are tied together by molecules running through more than one crystal and are known as intercrystalline links [17,43]. PGSu in a low concentration (PLCL2 and PLCL5) of molecules might play a role in intercrystal linking molecules as entanglements in amorphous polymers.

All bicomponent fibres were found as nontoxic in vitro. This allows us to conclude that PGSu may be an efficient eco-friendly plasticiser, changing the mechanical properties of selected polyesters and an accelerant of polymeric degradation. The second role is important in case of the slow degradation rate of polyesters in vivo [6,19,44]. This also makes it a promising candidate as a plasticiser in the food packaging industry. Since both glycerol and succinate acid are on the FDA’s generally recognised as safe (GRAS) list, the degradation of HBPEs does not pose a threat to the environment [45].

## 5. Conclusions

Our data suggested that the eco-friendly polymeric component changes chain mobility and influences the structure and mechanical properties of electrospun fibres. Evidence of the chemical groups, molecular mass of the polymer, and primary molecular structure determine the direction of changes. We believe that the results obtained in this work provide some new insights into the understanding of the effect of poly(glycerol succinate) on molecular mobility and the structural development of amorphous and semicrystalline polymers and contributes conscious use as well. In the future, it would be very interesting to investigate the biodegradation behaviour of the analysed compositions and the response of surrounding tissues under in vivo conditions.

## Figures and Tables

**Table 1:** Samples acronyms. PLCL: poly (L-lactide-co-caprolactone), PLLA: poly(L-lactic acid).

Sample Acronym with PLLA	Sample Acronym with PLCL	Main Polymer Amount % (w/w)	PGSu Amount % (w/w)
PLLA0	PLCL0	100	0
PLLA2	PLCL2	98	5
PLLA5	PLCL5	95	5
PLLA10	PLCL10	90	10
PLLA20	PLCL20	80	20
PLLA40	PLCL40	60	40

**Table 2:** Characteristic enthalpies and temperatures of bicomponent fibres with PGSu additive in relation to PLLA and PLCL determined from differential scanning calorimetry (DSC).

Sample Name	T_g_ of PGSu [°C] ^a^	T_g_ of PLLA [°C]	Enthalpy of Relaxation of PLLA [J/g]	Relaxation Temperature of PLLA [°C]	Cold Crystallisation [J/g]	Tc of PLLA [°C]	Enthalpy of Crystal Phase Melting [J/g]	T_m_ [°C]	Sample Name	T_g_ of PGSu [°C]	Enthalpy of Crystal Phase Melting [J/g]	T_m_ [°C]
**PLLA0**	^b^	50.87	15.99	55.32	13.64	67.57	45.02	180.80	**PLCL0**	-	24.89	157.24
**PLLA2**	-	50.84	10.65	56.6	16.71	66.78	53.54	182.66	**PLCL2**	-	24.93	156.98
**PLLA5**	−32.53	50.72	9.39	56.67	15.52	67.44	53.301	182.41	**PLCL5**	−15.98	25.26	157.78
**PLLA10**	−32.58	50.55	8.92	56.66	15.57	67.64	51.08	181.07	**PLCL10**	−20.56	29.66	157.04
**PLLA20**	−30.92	49.21	7.86	56.97	11.36	67.89	32.58	180.18	**PLCL20**	−25.20	31.45	157.11
**PLLA40**	−27.83	48.34	2.71	59.92	12.14	71.98	16.04	180.12	**PLCL40**	−30.96	27.04	156.28

^a^*T*_g_ of PGSu = −38.13 °C; ^b^ *T*_g_ of PGSu was not observed.
